# Farmers’ use and adaptation of improved climbing bean production practices in the highlands of Uganda

**DOI:** 10.1016/j.agee.2017.09.004

**Published:** 2018-07-01

**Authors:** E. Ronner, K. Descheemaeker, C.J.M. Almekinders, P. Ebanyat, K.E. Giller

**Affiliations:** a Plant Production Systems, Wageningen University, P.O. Box 430, 6700 AK Wageningen, The Netherlands; b Knowledge, Technology and Innovation, Wageningen University, P.O. Box 8130, 6700 EW Wageningen, The Netherlands; c International Institute of Tropical Agriculture, P.O. Box 7878, Kampala, Uganda

**Keywords:** *Phaseolus vulgaris*, Legumes, Co-design, Adoption, Smallholder, Nitrogen fixation, East Africa

## Abstract

Climbing beans offer potential for sustainable intensification of agriculture, but their cultivation constitutes a relatively complex technology consisting of multiple components or practices. We studied uptake of improved climbing bean production practices (improved variety, input use and management practices) through co-designed demonstrations and farmer-managed adaptation trials with 374 smallholder farmers in eastern and southwestern Uganda. A sub-set of these farmers was monitored one to three seasons after introduction. About 70% of the farmers re-planted climbing beans one season after the adaptation trial, with significant differences between eastern (50%) and southwestern Uganda (80–90%). Only 1% of the farmers used all of the improved practices and 99% adapted the technology. On average, farmers used half of the practices in different combinations, and all farmers used at least one of the practices. Yield variability of the trials was large and on average, trial plots did not yield more than farmers’ own climbing bean plots. Yet, achieved yields did not influence whether farmers continued to cultivate climbing bean in the subsequent season. Uptake of climbing beans varied with household characteristics: poorer farmers cultivated climbing beans more often but used fewer of the best-bet practices; male farmers generally used more practices than female farmers. Planting by poorer farmers resulted in adaptations such as growing climbing beans without fertilizer and with fewer and shorter stakes. Other relationships were often inconsistent and farmers changed practices from season to season. The diversity of farmer responses complicates the development of recommendation domains and warrants the development of a basket of options from which farmers can choose. Our study shows how adoption of technologies consisting of multiple components is a complicated process that is hard to capture through the measurement of an adoption rate at a single point in time.

## Highlights

• Climbing beans were more widely grown in southwestern than in eastern Uganda.
• 99% of the farmers adapted the best-bet climbing bean technology.
• On average, farmers used half of the improved climbing bean production practices.
• Use of practices by individual farmers was not consistent over time.
• Diversity in use of practices complicates measurements of adoption.

## 1 Introduction

The East African highlands are densely populated, and decreasing farm sizes and declining soil fertility status require agricultural intensification to sustain food production and avoid encroachment into forests (Benin et al., 2002, De Bauw et al., 2016, Sassen et al., 2013). The integration of legumes in farming systems provides a pathway for sustainable intensification of agriculture (Giller and Cadisch, 1995, Snapp et al., 2002b). Common bean is an important staple crop in many East African countries and a source of protein, calories, minerals and vitamins. Climbing beans offer potential to intensify bean production compared with bush beans, with yield potential being their greatest advantage: up to 4–5 t ha^−1^ (Checa et al., 2006) versus 1 to 2 t ha^−1^ for bush beans in Uganda (Kaizzi et al., 2012). Climbing beans are also more resistant to fungal and root rot diseases (Mcharo and Katafiire, 2014), and have a better potential to fix nitrogen (Bliss, 1993, Ramaekers et al., 2013, Wortmann, 2001). Improved varieties of climbing bean were introduced in Rwanda in the 1980s (Sperling and Muyaneza, 1995) and were rapidly adopted, particularly in the highlands of northern Rwanda. Climbing beans spread from Rwanda to neighbouring countries such as Burundi, DRC and Uganda in areas above 1600 m above sea level (masl) (Franke et al., 2016).

Climbing beans are not a simple replacement of bush beans as the latter are often intercropped with maize or grown as an understory in banana-coffee systems. Elsewhere, in Latin America, maize and climbing bean intercropping is common (Clark and Francis, 1985, Davis and Garcia, 1983), but in African systems where elevation is lower climbing beans grow too fast and smother the maize. Climbing beans are therefore better grown as sole crops. In addition, climbing beans need stakes to realize their climbing potential, implying additional costs for materials and labour. Moreover, because of their larger biomass production, climbing beans require more nutrient inputs (Sperling and Muyaneza, 1995). Altogether, adopting climbing beans constitutes a relatively complex change in farming practice and is not a mere replacement of cultivar.

Best yields of climbing bean are achieved through a combination of practices: the use of improved varieties, phosphate fertilizer and organic fertilizer, row planting, sole cropping, a high density of strong and tall stakes, timely planting and proper weeding (Franke et al., 2016, Kaizzi et al., 2012). Given the heterogeneity of African smallholder farming systems, these practices and their optimal combination (together representing the ‘climbing bean technology’) need to be tailored to fit the local agro-ecological, socio-economic and cultural environment (Descheemaeker et al., 2016, Giller et al., 2011). As argued for other complex technologies consisting of multiple components, it is unlikely that all farmers would adopt all components, or that adoption takes place as a simple, linear process (Brown et al., 2017, Glover et al., 2016).

In this study, we used the outcome of a co-design process with farmers, extension officers, NGOs and researchers to introduce improved climbing bean production practices among smallholder farmers in the highlands of eastern and southwestern Uganda. Farmers applied these practices on their own field in a so-called ‘adaptation trial’ and were monitored during and after the trial. Feedback from farmers’ experimentation and their adaptation of the technology, and understanding the reasons for (non-)use of practices in subsequent seasons provides insight in the adoption process and dynamics over time (Doss, 2006).

We also explored the relationship between the use of climbing bean production practices and a range of agro-ecological, plot and household characteristics. Variables selected were largely based on previous work on understanding the heterogeneity of African smallholder farming systems (Giller et al., 2011, Tittonell et al., 2005, Tittonell et al., 2010), and on adoption studies of agricultural technologies (Feder and Umali, 1993, Kassie et al., 2015, Knowler and Bradshaw, 2007) and legumes (Farrow et al., 2016). Agro-ecological characteristics are important to determine the biophysical relevance of technologies (Farrow et al., 2016). Plot characteristics such as land tenure, soil fertility and soil depth are often considered in relationship with the willingness to invest in improvement of the land (Banadda, 2010, Kassie et al., 2015, Kerr et al., 2007). Household characteristics (demographics, access to capital and labour, production orientation and importance of farm/off-farm income) define farmers’ ability to implement new technologies (Feder and Umali, 1993, Marenya and Barrett, 2007, Pircher et al., 2013). We also considered farmers’ previous experience with the technology, as decisions to use a certain practice may be related to earlier choices (Cowan and Gunby, 1996, Kassie et al., 2013).

Our objective was to understand the change in climbing bean production practices and the reasons for these changes among farmers of different geographical areas and socio-economic backgrounds, and to use this understanding to inform technology re-design and to delineate recommendation domains. We hypothesized that the majority of farmers would not adopt all components of the climbing bean technology, and that the use of practices would be related to performance of the adaptation trial, household wealth and farmers’ previous experience with the practices.

## 2 Methodology

### 2.1 Study area

The study was conducted in Kapchorwa District in eastern Uganda, located between 34.30° and 34.55° East and 1.18° and 1.50° North, and Kabale and Kanungu Districts in southwestern Uganda, located between 29.60° and 30.30° East and 0.35° and 1.50° South. The study sites are situated in the highland areas of Uganda, around 1800–1900 masl (Table 1). Both have two rainy seasons per year, and average annual rainfall in Kapchorwa district is 400–500 mm more than in the other two districts. Other important differences between the districts include soil type (of volcanic origin in Kapchorwa district and parts of Kanungu district, and Acrisols in Kabale district), market access, population density and experience with climbing bean cultivation, although the latter also differs within districts.

**Table 1:** Characteristics of study sites in eastern and southwestern Uganda.

	Southwestern Uganda		Eastern Uganda
District	Kabale	Kanungu	Kapchorwa
Elevation (masl)	1800	1850	1900
Rainfall (mm)^a^	1100	1200	1600
Cropping season A	Feb-Jun	Feb-Jun	Mar-Jul
Cropping season B	Aug-Nov	Aug-Nov	Sep-Dec
Soil type^b^	Acrisols	Acrisols/Andosols	Andosols
Distance to main market	Medium: 1.5 to 2 h (dirt road)	Poor: 2.5 to 3 h (dirt road)	Good: 1 to 1.5 h (tarmac road)
Population density (people km^−1^)^c^	207	57	297
Experience climbing bean cultivation	Medium	Long	Short

a climate-data.org.

b www.soilgrids.org.

c www.ubos.org.

### 2.2 Dissemination of the climbing bean technology

The study was conducted in the context of the N2Africa project. The climbing bean technology (combination of improved variety, input use and management practices) was disseminated in the format of ‘mother and baby trials’ (Snapp, 2002), whereby a large demonstration plot facilitated learning and comparison of a range of treatments throughout the season, and small trials enabled the testing of one treatment on farmers’ fields. In this study we call these ‘demonstration’ and ‘adaptation’ trials respectively.

***Demonstration trials*** showed a number of varieties, inputs, staking methods and other agronomic management practices. Treatments for these demonstration trials were developed in a co-design process with farmers, researchers, extension officers and NGO staff over a total of four seasons in 2014 and 2015 (see Descheemaeker et al. (2016)). The demonstrations started with a number of practices distilled from researchers’ experiences. Farmers evaluated the practices, which served as input for a re-design session with all stakeholders in which practices were modified, added or discarded to develop a ‘basket of options’ (Giller et al., 2011). Treatments in the demonstration therefore varied over locations and seasons (Supplementary material, Table S1). However, every season it was ensured that a ‘researcher best-bet’ and a control treatment were included.

We defined the *researcher best-bet technology* as the combination of practices that is expected to give the best climbing bean yield, and which was based on previous research on legumes in general and climbing beans specifically by Uganda’s National Agricultural Research Organisation (NARO) and project staff. The researcher best-bet technology consisted of the following components: an improved climbing bean variety with cattle manure and Triple Super Phosphate (TSP), planted as sole crop and in rows spaced at 50 cm between rows and 25 cm between plants, 2 seeds per hole (i.e. a density of 160,000 plants per ha), 40,000 stakes per ha and stakes taller than 1.75 m. The control treatment had the same variety and management practices but was planted without manure and TSP. The researcher best-bet and the control both had single, wooden stakes.

Because climbing beans were new for many farmers in Kapchorwa and poor availability of stakes due to deforestation was mentioned as important constraint for the cultivation of climbing beans, a low-cost and environmentally sustainable alternative in the form of strings from sisal, banana fibre or papyrus was offered in the demonstrations. Tripods (three wooden stakes tied together) were expected to enhance yields and were included in the demonstrations in Kapchorwa as well.

For the ***adaptation trials*** farmers received a package of seed of an improved climbing bean variety and TSP fertilizer at the equivalent of 15 kg P ha^−1^. An instruction leaflet with directions for planting and a number of best management practices was handed out together with the package, but farmers planted the package without further assistance. Adaptation trials were planted in seasons 2014B, 2015A and 2015B. In season 2014B, the leaflet instructed to plant two plots of 5 × 5 m: a plot with climbing bean variety NABE 26C with TSP, and a control plot with the same variety without TSP. Seeds for the two plots and TSP for one plot were provided in the package. Planting (spacing, density, sole or intercropping), staking (method, material) and weeding (timing, frequency) was left to the farmers (i.e. the leaflet specified that farmers could plant the way they normally do). In seasons 2015A and 2015B, farmers received inputs for one climbing bean plot only. Farmers could choose from a number of varieties, and about 80% received TSP fertilizer as well (based on availability). The idea of a control plot was abandoned in these seasons, as only few farmers had planted a comparable control plot previously. Instead, farmers were encouraged to compare the package with the way they normally grow climbing beans, and hence to plant the adaptation trial next to their own climbing bean variety with the practices they would normally apply. The plots could therefore differ with respect to multiple practices. In 2015, best practices for planting (plant spacing, number of seeds per hole) and staking were also included in the instruction leaflet. We will refer to the ‘N2Africa plot’ planted with the seed and fertilizer provided for the adaptation trial, the ‘control plot’ without TSP in 2014B and the ‘own climbing bean plot’ in 2015A&B.

### 2.3 Data collection

#### 2.3.1 Monitoring of adaptation trials

In seasons 2014B, 2015A and 2015B a stratified, random sub-set of farmers who planted an adaptation trial was monitored. Stratification was based on the variety received, with a minimum of five farmers per variety per district. The campaign started in 2014B in Kapchorwa District, eastern Uganda. From 2015A onwards, Kabale and Kanungu Districts were included. Over the three seasons, a total of 374 farmers from which 235 in Kapchorwa, 71 in Kabale and 68 in Kanungu were monitored (Table 2). Monitoring took place through a survey, tablet-based and programmed in ODK software (https://opendatakit.org/). The survey was conducted among the farmers who implemented the trial. If this person was not around, the household was not surveyed and the next household on the list with the same variety was sampled. The survey consisted of two parts: the first part was conducted in the field, before harvest. This part contained questions related to planting of the package, implementation of management practices and reasons for (not) applying these practices. The survey also contained questions on the characteristics of the field in which the trial was planted, and a number of questions on household characteristics. Field measurements (size of the N2Africa and own plot, plant density, stake density and length, etc.) were also taken.

**Table 2:** Total number of farmers participating in adaptation trials, number of farmers monitored and harvest data available for farmers in Kapchorwa, Kabale and Kanungu districts in seasons 2014B, 2015A and 2015B.

		2014B	2015A	2015B	Total
Kabale	Farmers participating	–	68	51	119
	Farmers monitored	–	41	30	71
	Farmers with harvest data	–	22	10	32

Kanungu	Farmers participating	–	100	106	206
	Farmers monitored	–	34	34	68
	Farmers with harvest data	–	20	21	41

Kapchorwa	Farmers participating	271	399	304	974
	Farmers monitored	88	88	59	235
	Farmers with harvest data	19	42	25	86

#### 2.3.2 Measurements of climbing bean yield

For the second part of the survey after harvest, farmers with two clearly distinguishable plots suitable for harvest measurements (i.e. plots planted in the same or a nearby field, at more or less the same time (average difference was 4 days)) were selected. Questions were asked on the inputs applied, the timing of management practices and problems (pest, disease, drought, waterlogging, etc.) encountered during the season. Farmers evaluated the performance of the trial and their own climbing bean plot. The bean harvest of the two total plots was measured with a digital scale as shelled or unshelled, according to how the farmer harvested the beans. In some cases the own plot was too large to harvest in total and a smaller harvest area was measured, representative for the field and easy to delineate for the farmer. Unshelled yields were converted to shelled yields with a factor of 0.7 based on earlier trials (no difference between varieties). Farmers also recorded if they had already sold or consumed part of the harvest. This amount was added to the measured grain weight.

#### 2.3.3 Use of practices in the season(s) after the adaptation trial

Another follow-up survey was carried out in the season after farmers participated in the adaptation trials. This survey aimed to assess the cultivation of climbing beans and the use of practices with farmers’ own seeds and inputs one season after participation in the trial. The follow-up survey was conducted among a random sub-sample of the farmers who were monitored during the adaptation trials. Again, the farmer who was responsible for the implementation was surveyed. This survey was carried out in seasons 2015A and 2015B in Kapchorwa, and in 2015B in Kabale and Kanungu (Table 3A) among a total of 148 farmers. The survey contained questions related to the practices shown in the demonstration trial in the previous season, to what extent these practices were new for farmers, and if farmers currently cultivated climbing beans with their own seed and used any of the previously demonstrated practices. The survey also contained open questions related to the reasons for (non-)use of any of the practices.

**Table 3:** Sub-set of farmers monitored one season after participation in adaptation trials in Kapchorwa, Kabale and Kanungu districts in seasons 2015A and 2015B (*n* = 148 unique farmers) (A), and sub-set of farmers monitored for multiple seasons in Kapchorwa district in seasons 2014A, 2015A and 2015B (B). Arrows indicate sub-sets of the same farmers that were monitored for one (arrow 1), two (arrows 2 and 3) or three seasons (arrow 4) after the adaptation trials.

Among the 29 farmers who participated in the follow-up survey in Kapchorwa in 2015A (Table 3A and B, arrow 1), a random subset of 20 farmers was monitored for a second season in 2015B (Table 3B, arrow 2). In addition, the survey was conducted in Kapchorwa in 2014A, among 43 farmers (Table 3B) who participated in earlier climbing bean trials in seasons 2013A and 2013B. A random sub-sample of these 43 farmers was also monitored for a second season (30 farmers, Table 3B, arrow 3), and again a sub-sample of these 30 farmers (those who could be traced back) for a third season (20 farmers, Table 3B, arrow 4). This made it possible to track the use of practices over time among the same group of farmers. These farmers, monitored for more than one season in Kapchorwa district only, were treated as a separate group within the study (Section 3.4).

### 2.4 Data analysis

#### 2.4.1 Measuring use, non-use and adaptation

We used the framework for measurements of adoption of complex technologies by Brown et al. (2017) to define use, non-use and adaptation of the researcher best-bet technology. The researcher best-bet technology consisted of a combination of individual practices. For each individual practice we measured if farmers used the practice or not as a binomial variable (use or non-use) according to the criteria specified in Table 4. Farmers who used all individual practices were considered to use the full researcher best-bet technology. Farmers who used none of the practices were non-users of the technology. Farmers who used a selection of practices were considered to modify the technology (did not use the technology to the full threshold, cf. Brown et al., 2017). We called this an adaptation of the researcher best-bet technology. For varieties specifically, we also measured if farmers completely replaced their old variety, or if they grew the improved variety next to their old variety. The latter was defined as partial use (i.e. the new practice has not completely replaced the old practice). Over time, farmers could move between different stages: from adaptation to use or from use to non-use or adaptation.

**Table 4:** Criteria used to define use, non-use and adaptation of the researcher best-bet technology and the individual practices composing this technology by farmers during and one or more seasons after participating in the adaptation trials.

Individual practices and researcher best-bet technology	Definition
*Individual practices*	
Improved variety	Use = planted variety from adaptation trial package
	Non-use = planted different variety than provided in the adaptation trial package
TSP	Use = applied TSP fertilizer
	Non-use = applied no fertilizer or a different type of fertilizer (DAP, NPK)
Organic fertilizer	Use = applied animal manure, crop residues, household waste
	Non-use = applied no organic fertilizer
Sole cropping	Use = applied sole cropping
	Non-use = applied intercropping
Row planting	Use = applied row planting
	Non-use = applied random planting, broadcasting
Seeds per hole	Use = applied 2 seeds per hole
	Non-use = applied 1 or >2 seeds per hole
Plant density	Use = applied 144,000 to 176,000 plants per ha (160,000 plants plus or minus 10%)
	Non-use = applied <144,000 or >176,000 plants per ha
Plants per stake	Use = applied ≤4 plants per stake
	Non-use = applied >4 plants per stake
Stakes per ha^a^	Use = applied 36,000 stakes per ha or more (40,000 stakes minus 10%)
	Non-use = applied <36,000 stakes per ha
Stake length^a^	Use = applied an average stake length ≥ 1.75 m
	Non-use = applied an average stake length <1.75 m

*Researcher best-bet technology*	Use = applied all individual practices
	Non-use = did not use any of the individual practices
	Adaptation = applied a selection of individual practices

a Practice only measured in season of adaptation trial, not in season after.

#### 2.4.2 Statistical analyses

Statistical analyses were performed in RStudio version 1.0.143 (R Core Team, 2017). Differences in climbing bean grain yield and the effect of the use of individual practices, planting dates and farmers’ estimated soil fertility on grain yield (Section 3.3.1) were analysed with a linear mixed model with district, season and plot as fixed and farm as random factor. Grain yields were square root transformed to ensure normality of residuals. Two outliers of yields of >8000 kg ha^−1^ on N2Africa plots were removed. Number of seeds per hole, plant density, stake density, number of plants per stake and stake length were assessed as numerical variables in this case and square root transformed to allow comparison between variables measured at different scales. The package *lmerTest* was used to detect significant differences, with an F-test for the fixed and a Likelihood Ratio Test for the random effects.

Linear models with season and district included as explanatory variables were used to assess the relationship between the total number of practices used per plot and climbing bean grain yield; yields in the adaptation trial and the use of practices one season after the trial; and farmers’ evaluation of the N2Africa and own climbing bean plot (measured on a scale from 1 (not satisfied at all) to 5 (very satisfied)) and the use of practices in the season after the adaptation trial.

Planting of climbing beans and the use of practices during and one season after the adaptation trial (Table 4) were related to a range of explanatory variables through univariate probit analyses (Section 3.3.2). Although the decision to use a certain practice may be related to the use of another practice and a multivariate probit would be more suitable to model such interrelated decisions (Kassie et al., 2013, Marenya and Barrett, 2007), our data was too unbalanced to result in useful outcomes. Instead, we assessed the correlation between practices separately to describe complementarity (positive correlation) or substitution (negative correlation) between practices. Generalized linear models with a probit link function were used for each individual practice. The function *step* with forward selection of variables was used to obtain a model per practice. Explanatory variables consisted of season, district, household, plot and agro-ecological characteristics. An overview and descriptive statistics of the explanatory variables are presented in Table 5. Livestock ownership was converted to Tropical Livestock Units (TLU) (Jahnke et al., 1982). Outliers of 15 and 20 TLU and farm sizes of 20 ha were removed. Farm size, TLU number of household members and age of the household head were square root transformed to ensure normality of residuals. Household characteristics were available for farmers during and after the trials, but plot characteristics only for the adaptation trials. We therefore considered two different models for the adaptation trials: one for household characteristics only (for comparison with the season after the adaptation trial), and one for the combination of all variables.

**Table 5:** Definitions and summary statistics (mean and standard deviation) of agro-ecological, plot and household characteristics during (*n* = 374) and one season after (*n* = 148) the adaptation trials per district.

	Adaptation trial				One season after adaptation trial			
	Kabale (n = 71)	Kanungu (n = 68)	Kapchorwa (n = 235)	Average (n = 374)	Kabale (n = 25)	Kanungu (n = 34)	Kapchorwa (n = 89)	Average (n = 148)
*Household characteristics*								
Number of household members	5.5 (2.3)	5.6 (2.3)	6.7 (2.9)	6.3 (2.8)	6.9 (2.4)	5.8 (1.8)	6.4 (3.2)	6.3 (2.8)
Gender of farmer (0 = female, 1 = male)	0.3 (0.5)	0.3 (0.5)	0.4 (0.5)	0.3 (0.5)	0.4 (0.5)	0.2 (0.4)	0.3 (0.5)	0.3 (0.5)
Gender of household head^a^ (0 = female, 1 = male)	0.9 (0.3)	0.9 (0.3)	0.9 (0.3)	0.9 (0.3)	1.0 (0.0)	1.0 (0.0)	0.9 (0.3)	0.9 (0.3)
Age household head	47 (14)	44 (15)	47 (15)	46 (15)	42 (12)	43 (14)	48 (16)	46 (15)
Education household head (0 = none or primary, 1 = secondary or higher)	0.2 (0.4)	0.1 (0.3)	0.6 (0.5)	0.5 (0.5)	0.2 (0.4)	0.2 (0.4)	0.6 (0.5)	0.5 (0.5)
Highest education in household (0 = none or primary, 1 = secondary or higher)	0.6 (0.5)	0.3 (0.4)	0.9 (0.4)	0.7 (0.5)	0.7 (0.5)	0.5 (0.5)	0.9 (0.3)	0.8 (0.4)
Farm size (ha)	0.6 (0.6)	1.7 (1.5)	1.0 (1.2)	1.1 (1.5)	0.9 (0.7)	2.4 (3.8)	1.2 (1.4)	1.5 (2.3)
TLU	1.3 (0.9)	0.8 (1.1)	1.7 (1.3)	1.5 (1.3)	2.2 (4.9)	1.0 (1.3)	2.3 (2.2)	2.0 (2.5)
Months food secure (0 = <10 months, 1 = 10–12 months)	0.7 (0.5)	0.8 (0.4)	0.4 (0.5)	0.5 (0.5)	0.5 (0.5)	0.6 (0.5)	0.6 (0.5)	0.6 (0.5)
Production orientation (0 = all or most farm produce consumed, 1 = half or most farm produce sold)	0.5 (0.5)	0.1 (0.3)	0.5 (0.5)	0.4 (0.5)	0.6 (0.5)	0.1 (0.3)	0.5 (0.5)	0.4 (0.5)
Off-farm income (0 = all or most income from farming, 1 = half or most income from off-farm activities)	0.1 (0.3)	0.4 (0.5)	0.2 (0.4)	0.2 (0.4)	0.1 (0.4)	0.2 (0.4)	0.3 (0.4)	0.2 (0.4)
Frequency of hiring labour (0 = never or sometimes, 1 = regularly or permanently)	0.3 (0.5)	0.5 (0.5)	0.4 (0.5)	0.4 (0.5)	0.7 (0.5)	0.8 (0.4)	0.6 (0.5)	0.7 (0.5)
Farmers with income from working on other people's fields (0 = no, 1 = yes)	0.0 (0.2)	0.2 (0.4)	0.2 (0.4)	0.2 (0.4)	0.0 (0.0)	0.1 (0.3)	0.1 (0.3)	0.1 (0.3)
Farmers with income from salary, pension or remittances (0 = no, 1 = yes)	0.1 (0.3)	0.1 (0.3)	0.3 (0.4)	0.2 (0.4)	0.1 (0.3)	0.1 (0.4)	0.2 (0.4)	0.2 (0.4)

*Plot and agro-ecological characteristics*								
Land tenure (1 = owned, 0 = otherwise)	0.9 (0.3)	0.9 (0.3)	0.9 (0.4)	0.9 (0.3)	–	–	–	–
Farmer perceived soil fertility (3 = good, 2 = moderate, 1 = poor)	2.3 (0.7)	2.4 (0.8)	2.5 (0.7)	2.4 (0.7)	–	–	–	–
Farmer perceived soil fertility of plot in relation to other plots (3 = better, 2 = same, 1 = poorer)	2.0 (0.8)	2.2 (0.9)	2.2 (0.7)	2.2 (0.8)	–	–	–	–
Soil depth measured up to 50 cm	34 (9)	39 (7)	47 (5)	43 (8)	–	–	–	–
Elevation (masl)	1792 (61)	1857 (105)	1862 (105)	1848 (102)	–	–	–	–
Availability of trees for staking	2.3 (0.5)	2.6 (0.5)	2.1 (0.2)	2.2 (0.4)	–	–	–	–

a Farmers reported if they were head of the household or not.

Finally, univariate probit models per practice (season and district included as explanatory variables) were used to relate planting of climbing beans and the use of practices in the season after the adaptation trial to previous experience with the cultivation of climbing beans (had farmer ever grown climbing beans before) and the use of practices (had farmer ever used the practice in climbing bean before) (Section 3.3.3).

## 3 Results

### 3.1 Use and adaptation during and one season after the adaptation trials

#### 3.1.1 Climbing bean cultivation and use of practices

**Fig. 1:**
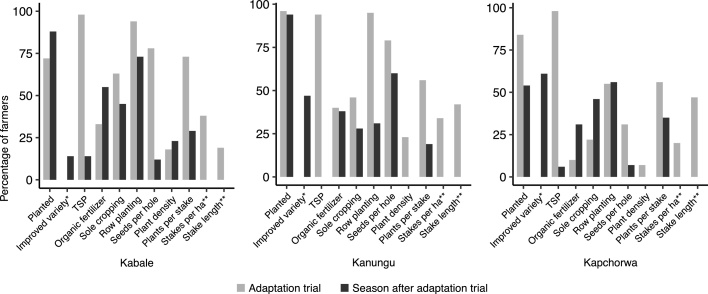
Percentage of farmers planting seed from package and using demonstrated climbing bean practices in adaptation trials (seasons 2014B, 2015A and 2015B) (n = 374) and one season after the adaptation trials with their own seeds and inputs (seasons 2015A and 2015B) (*n* = 148) in Kabale, Kanungu and Kapchorwa districts in Uganda. *Planting an adaptation trial by definition meant planting the variety distributed in the adaptation trial package. The percentage of farmers using the improved variety in the adaptation trial is therefore not indicated (100% by default). **The number of stakes per ha and stake length were only assessed in the season of the adaptation trial.

About 85% of the farmers who received seed of an improved climbing bean variety for an adaptation trial planted the seed (Fig. 1). Most non-planters said they would keep the seed for next season, a few farmers ate the seed or gave it away. One season after the adaptation trial, 70% of the farmers re-planted climbing beans. There were large differences between districts, however: 90–95% of the farmers in Kabale and Kanungu planted, but only 50% in Kapchorwa. About 50–60% of the farmers who planted climbing beans in the season after the adaptation trial chose to grow the same improved variety as they received for the trial, except in Kabale where this was only 14% (3 farmers). Most farmers in all three districts who continued to cultivate the improved variety after the trials grew this variety next to their old variety (partial use).

About 80% of the farmers who planted (*n* = 251) received TSP as part of the adaptation trial. All but three farmers used the TSP, and another six farmers used only part of the TSP because they applied it on another crop or saved it for next season. One season after the adaptation trial the use of TSP fertilizer fell to only three farmers in Kabale and three farmers in Kapchorwa. Five out of these six farmers did not plant in the previous season and simply used the TSP that was provided in the adaptation trial. Therefore overall only one farmer in Kapchorwa bought TSP from an agro-dealer.

The use of organic fertilizer in the adaptation trials ranged from 10% in Kapchorwa to 40% in Kanungu. In the season after the trial the use of organic fertilizer increased in Kabale and Kapchorwa and remained more or less the same in Kanungu. The other management practices were generally implemented among a larger percentage of farmers in Kabale and Kanungu than in Kapchorwa during the season of the adaptation trial. In the season after the adaptation trial the differences between districts were less pronounced. The number of seeds per hole and plants per stake used by farmers were often larger than those demonstrated. Plant densities were smaller among farmers in Kanungu and Kapchorwa, but much larger in Kabale (average of 235,000 plants per ha).

Two of the management practices, stakes per ha and stake length, were only assessed during the adaptation trials and not in the season after. The demonstrated number of stakes per ha was used by 25% of the farmers. The average ranged between 27,000 stakes per ha in Kapchorwa and 34,000 stakes per ha in Kanungu. An average stake length of 1.75 m or more was only used by about 20% of the farmers in Kabale, and by 40–50% of the farmers in Kanungu and Kapchorwa. The average stake length in Kabale was 1.60 m, in Kanungu and Kapchorwa 1.74 m.

#### 3.1.2 Researcher best-bet technology

During the adaptation trials, only two (out of 177) farmers used all seven practices of the best-bet technology that were monitored during and after the adaptation trial (TSP, organic fertilizer, sole cropping, row planting, seeds per hole, plant density and plants per stake) (Fig. 1). Hence, all other farmers (99%) adapted the technology, yet none of the farmers used none of the practices. The average number of practices used was 3.8 and was largest in Kanungu (4.4), followed by Kabale (4.2) and Kapchorwa (3.5). If we also consider the stakes per ha and stake length, none of the farmers used the full researcher best-bet technology. In the season after the adaptation trial, the average number of practices decreased to 2.8 (2.9 in Kabale, 3.6 in Kanungu and 2.4 in Kapchorwa), but again all farmers used at least one of the practices

### 3.2 Reasons for use and adaptation

#### 3.2.1 Climbing bean cultivation

The farmers who continued the cultivation of climbing beans in the season after the adaptation trial largely mentioned good yields as positive aspect of climbing beans (80% in Kabale, 50% in Kanungu and 40% in Kapchorwa). Farmers who did not grow climbing beans after the adaptation trial mostly mentioned poor weather conditions (too much rainfall or sunshine) (32%), a lack of stakes (29%), or a lack of seed due to poor yields in the previous season or destruction of the seed during storage (27%). Almost 70% of the farmers who did not plant climbing beans in the season after the adaptation trial grew bush beans instead. Main reasons to grow bush beans instead of climbing beans were that bush beans do not require stakes (55%), and bush beans were perceived to be more tolerant to sunshine than climbing beans (32%).

#### 3.2.2 Use of practices

Farmers who continued the cultivation of the distributed varieties often mentioned the good yield and taste of these varieties. Farmers who cultivated the new variety next to their old variety (partial use) did this because the old variety had a ready market, a good taste, the seed was more easily available (in large quantities), or the variety was more tolerant to the prevailing weather conditions. Main reasons to reject the distributed variety were the better yield (34%), market prices (19%), and tolerance to weather conditions (19% of the farmers in Kanungu) of their old variety. In Kapchorwa, farmers using either the distributed or their old variety mentioned that this variety was the only seed available.

A very common adaptation of the researcher best-bet technology was to grow climbing beans without TSP or with a different type of P-fertilizer in the season after the adaptation trial. In Kapchorwa, about 30% of the farmers used DAP instead of TSP. DAP is extensively used for maize production in the area and is widely available. The use of DAP in bean (bush or climbing) was therefore already common practice among farmers in Kapchorwa, whereas TSP was not easily available at the time of study. Farmers who did not use P-fertilizer said the soil was already fertile or that fertilizer was too expensive. Organic fertilizer was applied by about half of the farmers. The others mentioned that the soil was already fertile (36%), that their fields were far away and transport of organic fertilizer is heavy (28%), or that organic fertilizer was not available (26%).

Another adaptation, practiced by the majority of farmers in Kanungu and Kapchorwa, was to grow the climbing beans in intercropping with (coffee and) banana instead of sole cropping. A few farmers in Kanungu intercropped with maize. The main reason for intercropping was a shortage of land (mentioned by 55% and 82%, respectively). Farmers who grew beans as sole crops generally did this to get good yields and to avoid competition for water, nutrients and light from other crops. For the adaptation trials specifically, farmers mentioned that sole cropping was taught in the demonstrations (22%) or that they wanted to see how the variety would yield when grown alone (19%).

In Kapchorwa, about half of the farmers planted in rows, and in Kabale and Kanungu row planting decreased considerably in the season after the trial. The main reasons given for random planting or broadcasting were tradition, a lack of time and labour, and ease of the method. Farmers also mentioned that they had to plant in between another crop that was already there. Farmers who planted in rows mentioned that this made management (staking, weeding, spraying) easier, gave better yields, or required fewer seeds than broadcasting. During the adaptation trials, 41% mentioned that row planting was taught in the demonstration or instruction leaflet.

Also farmers who planted two seeds per hole said they learned this in the demonstration (50%). Other reasons for reducing the number of seeds per hole were to avoid congestion or competition for nutrients and sunlight, or to plant a larger area. Farmers who planted a larger number of seeds per hole mentioned tradition, increasing the chances of plant survival and being efficient with the stakes. The latter was therefore also mentioned by farmers who applied more than four plants per stake. More than half of the farmers mentioned, however, that they just placed stakes randomly, and whatever number of plants that could reach the stake would climb on it. A shortage of stakes and tradition were also mentioned. Only 10% of the farmers in Kabale and Kanungu and 35% of the farmers in Kapchorwa ever selected stakes based on their length. Others referred to the shortage of stakes and just used whatever they could get (80%), mentioned that selecting long stakes was time consuming, or saw no specific reason to select long stakes.

#### 3.2.3 Staking of climbing beans

As the lack of stakes was mentioned as important constraint for climbing bean cultivation and alternative staking methods were offered in the demonstration, staking methods received specific attention. During the adaptation trials, single stakes were the most commonly used method by far because of tradition, the ease of the method the cost and availability of the material and a lack of knowledge of other methods. Seven farmers in Kapchorwa used tripods (of which four in combination with single stakes) because tripods were considered stronger than single stakes or as support for weaker stakes. One farmer in Kabale used sisal strings but commented that this was “way too tiresome” and he would not use them again. Five farmers did not stake at all due to illness, a lack of time, or destruction of the beans by cows roaming through the field.

We expected an increase in the use of the staking alternatives in the season after the trial, as 30–60% of the farmers indicated that they had then seen the alternatives in the demonstration trials. All farmers used single stakes, however, in the season after the adaption trial.

### 3.3 Explaining diversity in climbing bean cultivation and use of practices

#### 3.3.1 Performance of adaptation trials

##### 3.3.1.1 Climbing bean grain yield in adaptation trials

**Fig. 2:**
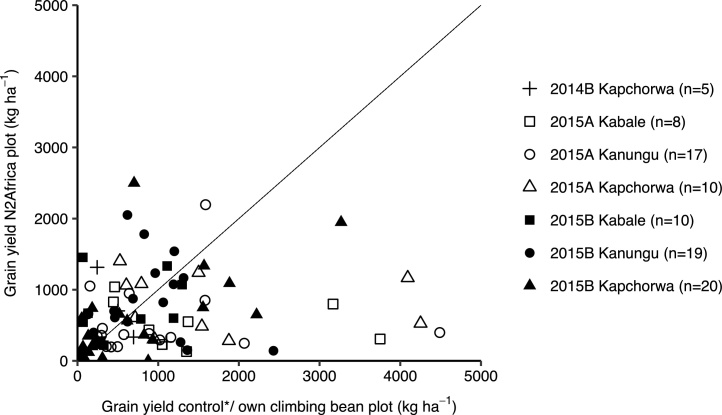
Paired observations of climbing bean grain yield (kg ha^−1^) on control (2014B) or own (2015A&B) climbing bean plot versus N2Africa plots per season and district. *N2Africa plots (with TSP) were compared with a control plot (without TSP) in season 2014B. In 2015A&B farmers planted an N2Africa plot next to their own climbing beans instead of a control plot.

Good or poor yields obtained in the adaptation trials were reasons mentioned by farmers to (dis)continue the cultivation of climbing beans. Climbing bean grain yields on the N2Africa and own climbing bean plot showed a large variation (Fig. 2). Some farms had very small yields on both plots, whereas others achieved yields of around 2500 kg ha^−1^. Especially in Kanungu in season 2015B the N2Africa plots seemed to perform better than own climbing bean plots, but there were many cases in which the N2Africa plot performed worse than the farmers’ own climbing bean plot. Average yields on the own climbing bean plot were therefore significantly larger than on the N2Africa plot in season 2015A (*P <* 0.05), but there were interactions between season and district (Table 6).

**Table 6:** Average grain yields (kg ha^−1^) of climbing bean on N2Africa and control or own plot in adaptation trials in seasons 2014B, 2015A and 2015B per district. Yields for each season + district combination were analysed separately in a linear mixed model with plot as fixed and farm as random effect, due to an interaction between season, district and yield.

	2014B			2015A			2015B		
	N2Africa	Control	*P*	N2Africa	Own	*P*	N2Africa	Own	*P*
Kabale	–	–	–	573	1236	ns	687	531	ns
Kanungu	–	–	–	545	965	ns	660	801	ns
Kapchorwa	284	513	ns	997	1686	<0.1	843	838	ns
Average	284	513	ns	816	1233	<0.05	739	769	ns

**Fig. 3:**
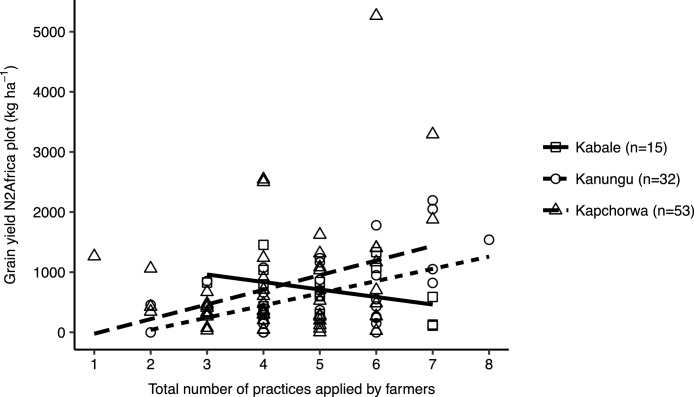
Relation between total number of practices applied (of TSP, organic fertilizer, sole cropping, row planting, seeds per hole, plant density, plants per stake, stakes per ha and stake length) and climbing bean grain yield (kg ha^−1^) on the N2Africa plot in adaptation trials per district. Relationship between yield and number of practices used only significant in Kanungu district (linear regression, *P < *0.01, *R^2^* = 0.23).

Although generally the N2Africa plots did not have better yields than the farmers’ own climbing bean plots, the total number of practices used on the N2Africa plot showed a positive relationship with climbing bean yields of these plots in Kanungu (*P <* 0. 05) and Kapchorwa (not significant) (Fig. 3). The number of stakes ha^−1^ was the only individual practice that had a highly significant positive effect on yield in all three districts (*P <* 0.001). Variety and row planting also tended to have an effect on yields (*P <* 0.1). Other practices such as TSP fertilizer or manure did not improve yields.

The general lack of difference or even better yields on the own climbing bean plot than on the N2Africa plot could have discouraged farmers to plant climbing beans in the season after the trial. However, yields of farmers who did and did not plant climbing beans one season after the trial did not differ. Conversely, better yields with more practices could have convinced farmers to use more practices in the season after the trial, but there was no relationship between yield during and the number of practices used after the trial.

The limited improvements and in some cases reduced yield on the N2Africa plot compared with the own climbing bean plot were not anticipated. In the demonstration trials in the same districts and seasons (data not presented), the combination of TSP and manure improved climbing bean yields (*P <* 0.05), and the increase in yields tended to be larger in improved than in local varieties (not significant). Manure and TSP only had positive effects as well, but these were not significant.

One explanation for the lack of yield improvement could be the late delivery of seed for the adaptation trial, often mentioned by farmers. More than a third of the farmers planted the N2Africa plot later than the own climbing bean plot, but there was no relationship between planting date and yield of the N2Africa plot or the own climbing bean plot. Planting dates were only available for 50% of the farmers, however, and not for Kapchorwa and Kabale (only 3 data points) in 2015A. In season 2015B (the season with the most data points available), there was a non-significant negative relationship between planting date and yield. Based on this, we cautiously conclude that the late arrival of seeds is one reason for the N2Africa plots performing worse than the own climbing bean plots that were planted earlier. Another reason could be that farmers decided to plant the N2Africa plot on relatively poorer fields than their own climbing beans. However, farmers’ indication of the (relative) fertility of the field had no effect on yield.

Farmers were also asked to explain the difference in yield between the two plots. Poor yields on the N2Africa plot were attributed to pests and diseases, weather conditions (too much rainfall, drought), damage by cows, goats or chickens and late planting. Good yields on the own climbing bean plot were often attributed to varieties: farmers’ own varieties were considered more resistant to weather conditions or pests and diseases. If yields on the N2Africa plot were larger, farmers mentioned the application of mineral or organic fertilizer and the use of other improved practices (number and length of stakes, row planting).

##### 3.3.1.2 Evaluation of adaptation trials

**Fig. 4:**
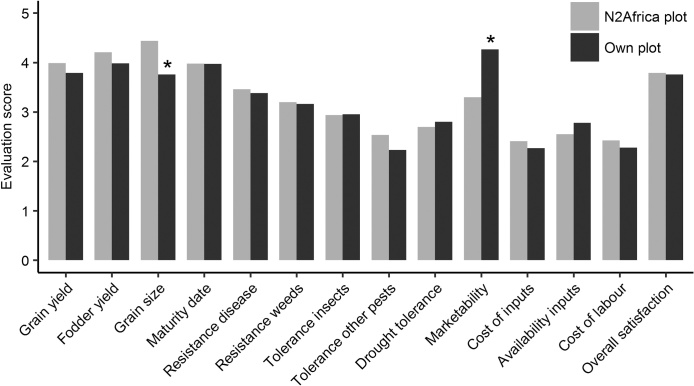
Farmers’ evaluation of the N2Africa plot and the own climbing plot in adaptation trials in seasons 2015A and 2015B (*n* = 152). Scores ranged from 1 (not satisfied at all) to 5 (very satisfied). * indicates significant difference in evaluation score between N2Africa and own climbing bean plot (One-way ANOVA, *P* < 0.05).

Farmers judged the trial and the different practices not only based on yields, but also on other aspects. In general, scores for the N2Africa plot were quite similar to the own climbing bean plot (Fig. 4). Grain size was the only aspect that scored better on the N2Africa than on the own plot (*P <* 0.05), although fodder yield and tolerance to pests other than insects tended to be better as well (*P <* 0.1). For marketability, the improved varieties scored worse than farmers’ own varieties.

The evaluations had limited predictive value for the use of practices in the season after participation in the trials. In general, farmers who planted climbing beans after the trials gave a significantly lower score for the marketability of the variety planted on the N2Africa plot than farmers who did not plant (*P <* 0.05). The farmers who continued cultivating the distributed varieties gave a significantly better score for the resistance to diseases (blight, anthracnose) of these varieties than farmers who did not plant. There were no significant relationships between the scores for costs and availability of inputs and the use of P-fertilizer.

#### 3.3.2 Household, plot and agro-ecological characteristics

Apart from performance of the trial, household characteristics were also expected to constrain or facilitate the cultivation of climbing beans and the use of practices. Planting of climbing beans during^1^ and after the adaptation trial showed a negative relationship with education of the household head; income from salary, pension or remittances and food security (Table 7). On the other hand, the relationship with farmers working on other people’s fields for income was positive. These variables are all proxies for farmers’ wealth, and suggest that planting was often done by poorer farmers. An exception was the positive relationship with the highest education level in the household.

**Table 7:** Coefficient estimates of household characteristics related to the use of practices during (*n* = 374) and one season after (*n* = 148) adaptation trials, tested with a univariate probit model and selected with the function *step*.

	Adaptation trials		One season after adaptation trials	
Planted^a^	Education hh head	−1.184*	District Kanungu	0.659
	Income casual labour on-farm	0.723†	District Kapchorwa	−0.798†
	Income salary/pension/remittances	−0.635†	Food security	−0.515†
	Highest education in household	1.251†		

Improved variety^b^	–		District Kanungu	0.450
			District Kapchorwa	1.252*
			Farm size	0.633†
			Highest education in household	−0.996*
			Season 2015B	−0.797†
			No of hh members	0.377

Organic fertilizer	District Kanungu	0.372	Season 2015B	1.443**
	District Kapchorwa	−1.055**	TLU	0.842**
	Gender of farmer	−0.900**	Age	−0.399*
	Farm size	0.540*		
	Gender hh head	−0.858*		
	Education hh head	0.501†		

Sole cropping	District Kanungu	−0.339	Season 2015B	−0.848*
	District Kapchorwa	−1.089**		
	Season 2015A	−0.643*		
	Season 2015B	−0.066		
	Hired labour	0.445*		

Row planting	Farm size^c^	1.116	Season 2015B	−11.000
			District Kanungu	−1.068*
			District Kapchorwa	−0.898†
			Gender hh head	5.243

Seeds per hole	District Kanungu	0.057	Season 2015B	2.096**
	District Kapchorwa	−1.438**	District Kanungu	1.591**
	TLU	0.569**	District Kapchorwa	0.944*
	Hired labour	−0.489*		

Plant density	Season 2015A	4.541	TLU	−0.463†
	Season 2015B	5.183		
	Farm size	0.599*		
	Gender hh head	0.710		

Plants per stake	Hired labour	−0.305†	Season 2015B	−1.657**
			Gender of farmer	0.813*
			Number of hh members	−0.855*
			TLU	0.702*

Stakes per ha^d^	Season 2015A	1.176**	–	
	Season 2015B	0.811*		
	Hired labour	−0.553*		
	Gender of farmer	0.405*		
	Off-farm income	0.459*		

Stake length^d^	Number of hh members	0.368*	–	
	Income salary/pension/remittances	0.458*		
	District Kanungu	0.704*		
	District Kapchorwa	0.519†		
	TLU	0.272		

*Note*: TSP was not considered as observations of farmers (not) applying TSP were too few.

a Household characteristics for farmers who did not plant the adaptation trial only available in season 2014B; in 2015A and 2015B only collected for farmers who planted the trial. Results presented for adaptation trials are for season 2014B only.

b All farmers who planted the adaptation trial used the variety distributed in the package, so explanatory variables for planting the trial and use of the improved variety are the same.

c (Almost) all farmers planted in rows in Kabale and Kanungu during the adaptation trials – results presented are for Kapchorwa only.

d Practice only measured in season of adaptation trial, not in season after.

† Significant difference *at *P* < *0.1.

* Significant difference at **P* < *0.05.

** Significant difference at **P* < *0.01.

Farmers in Kapchorwa planted climbing beans significantly less often in the season after the trial than in Kabale, but continued to grow the variety received in the adaptation trial package more often. The use of the improved variety was associated with larger farm sizes, but with poorer education of the household. TSP could not be considered as almost all farmers applied TSP during the adaptation trials, and almost none in the season after. Organic fertilizer was applied more often by female farmers, by farmers with larger farms and with better education. Livestock ownership and organic fertilizer were only positively related in the season after the trial. The other practices largely differed between seasons and districts. For instance, almost all farmers in Kabale and Kanungu planted in rows during the adaptation trials and only farmers in Kapchorwa planted randomly. In the season after the trial, all farmers in Kapchorwa in season 2015A planted in rows. In season 2015B results were mixed again in all three districts. Relationships with household characteristics were often inconsistent: the demonstrated number of seeds per hole was applied more often by farmers with more TLU, but less often by farmers who hired labour frequently. Likewise, plant density was positively related to farm size during the adaptation trial, but negatively with TLU in the season after. The relationship with gender of the farmer was often positive, meaning that male farmers generally applied more practices than female farmers.

Plot and agro-ecological characteristics (assessed in combination with household characteristics) also played a role in the use of most practices during the adaptation trials (data not presented). Organic fertilizer was applied to fields with larger soil depth (*P <* 0.1), sole cropping had a negative (*P <* 0.05), and the number of seeds per hole a positive (*P <* 0.1) relationship with ownership of the land, row planting (Kapchorwa district only) was mostly done at lower elevation (*P <* 0.01), and the demonstrated plant density was more often applied at higher elevation (*P <* 0.05). The number of stakes per ha and stake length were not related with any of the plot or agro-ecological characteristics. Only in the case of sole cropping, the selected plot and agro-ecological characteristics had a more significant contribution than household characteristics.

Farmers used several practices at the same time during the adaptation trials: there was a significant positive correlation between the use of organic fertilizer, sole cropping, row planting, the demonstrated number of seeds per hole and plant density (Table 8). In the season after the trial, however, row planting had a strong negative relationship with the number of seeds per hole and plant density. Observations of the latter practices were few, however. Farmers who planted the demonstrated number of seeds per hole in the season after the trial also continued planting of the improved variety and used TSP, sole cropping and row planting more often, but did not use the demonstrated number of seeds per hole and plant density.

**Table 8:** Correlation coefficients of use of climbing bean production practices during and one season after adaptation trials.

	Adaptation trials									One season after adaptation trials							
	TSP	OF	SC	RP	SEH	PD	PS	STH^b^	SL^b^	IV	TSP	OF	SC	RP	SEH	PD	PS
IV^a^										–							
TSP	–									0.18	–						
OF	0.08	–								−0.05	−0.01	–					
SC	0.06	0.31**	–							0.07	0.11	−0.10	–				
RP	−0.02	0.20**	0.31**	–						0.23*	0.19	−0.01	0.26**	–			
SEH	0.05	0.24**	0.23**	0.36**	–					−0.17	−0.09	−0.02	−0.19	−0.94**	–		
PD	0.06	0.24**	0.16*	0.14	0.19**	–				−0.13	0.14	−0.04	−0.15	−0.70**	0.67**	–	
PS	0.02	−0.05	−0.05	−0.05	0.24**	0.00	–			0.23*	0.29**	0.00	0.31**	0.58**	−0.55**	−0.30**	–
STH^b^	0.10	0.06	0.15	0.14	0.07	0.06	0.03	–									
SL^b^	0.06	−0.15*	−0.04	−0.08	−0.06	0.00	0.06	−0.04	–								

IV = improved variety, TSP = TSP fertilizer, OF = organic fertilizer, SC = sole cropping, RP = row planting, SEH = seeds per hole, PD = plant density, PS = plants per stake, STH = stakes per ha, SL = stake length.

a All farmers who planted the adaptation trial used the variety distributed in the package, so not considered for adaptation trials.

b Practice only measured in season of adaptation trial, not in season after.

* Significant difference at P* < *0.05.

** Significant difference at *P* < **0.01.

#### 3.3.3 Previous experience with the technology

**Fig. 5:**
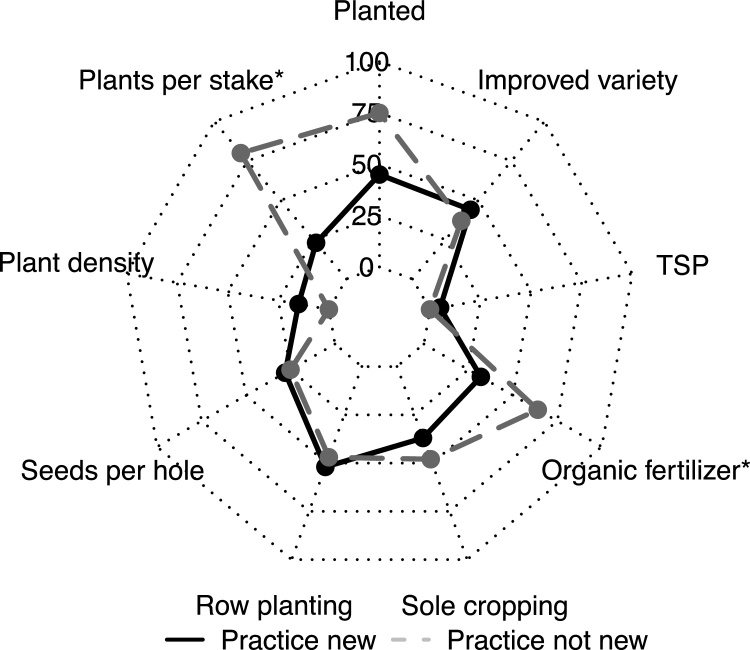
Percentage of farmers using individual practices one season after the adaptation trials with their own seed and inputs, by farmers for whom practice was new or not new when introduced in adaptation or demonstration trial (*n* = 148). Practices marked with * indicate significant differences (*P* < 0.05) between farmers for whom practice was (not) new (assessed with univariate probit model).

Farmers had different previous experience with climbing bean cultivation. All farmers in Kabale and Kanungu monitored one season after the adaptation trial indicated that they had ever grown climbing beans before, but in Kapchorwa only 70% of the farmers. The other practices were new to 50–100% of the farmers. Previous experience influenced the use of practices: farmers who had already used a practice in climbing beans before often used this practice more frequently than farmers for whom the practice was new (Fig. 5). Organic fertilizer and the demonstrated number of plants per stake were used significantly more often, and farmers who had already grown climbing bean before also tended to grow them more often than farmers for whom they were new. The latter were mainly the farmers in Kapchorwa.

### 3.4 Use and adaptation over time

**Fig. 6:**
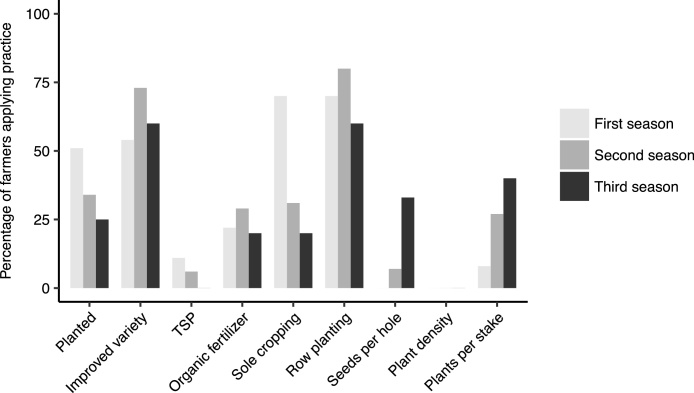
Subset of farmers in Kapchorwa district who planted climbing beans and applied individual practices one (*n* = 63), two (*n* = 50) and three (*n* = 20) seasons after participation in the adaptation trials (using their own seed and inputs).

Given that previous experience resulted in a more frequent use of practices, a consistent or even incremental use of practices over time was expected. A sub-group of farmers in Kachorwa was followed up to two (50 farmers) or three seasons (20 farmers) after the adaptation trial. These were mostly farmers from two sub-counties where climbing beans were not grown before (for 84% of farmers, climbing beans were new).^2^ About half of the farmers of this sub-group planted climbing beans in the first season after participation in the adaptation trials, but only 30% planted in the second season and 25% in the third season (Fig. 6). A lack of seed and drought were the most frequently mentioned reasons not to plant climbing beans. The use of the distributed varieties remained relatively constant at about 55–75%. The use of TSP decreased, but about 30% of the farmers in all three seasons used DAP. One farmer explicitly mentioned that he used DAP because TSP was not available. The percentage of farmers planting the beans as sole crops decreased from 70% to less than a quarter of the farmers over the seasons. The demonstrated number of seeds per hole and plant density were applied by very few farmers (the increase in the third season concerned only one out of three farmers with data for this variable), but the use of the demonstrated number of plants per stake increased over the seasons. All farmers in the first and second season used single staking, but in the third season one farmer used strings and indicated that this was due to a lack of stakes. The total number of best-bet practices applied remained stable between the first, second and third season after participation in the adaptation trial with an average of 2.2, 2.4 and 2.2 practices respectively, and none of the farmers used the full researcher best-bet.

**Fig. 7:**
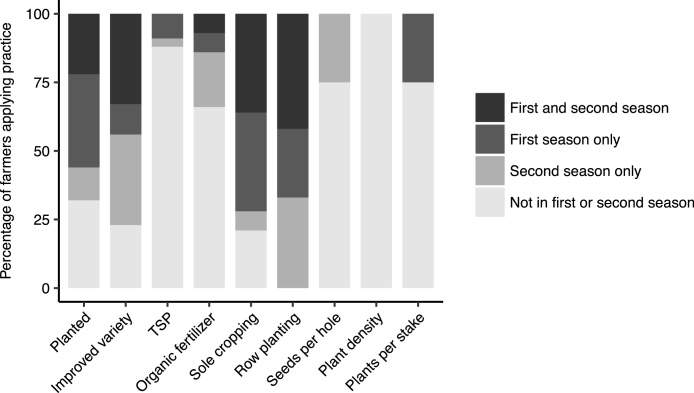
Subset of farmers in Kapchorwa district who were monitored for two seasons after the adaptation trials (*n* = 50), and percentage of these farmers who planted climbing beans and applied individual practices in the first and second, first or second, or none of the two seasons after participation in the adaptation trials (using their own seed and inputs).

The use of practices by individual farmers was not consistent over the seasons, i.e. the same farmer could use a practice during the first season, but not in the second or *vice versa*. From the 50 farmers that were monitored for two seasons, about a quarter of the farmers planted both in the first and second season, and about 50% planted in one of the two seasons (Fig. 7). TSP was not used in any of the seasons by about 90% of the farmers, and organic fertilizer by 75%. All farmers practiced row planting in one of the two seasons. The majority of farmers (75–100%) did not use the demonstrated number of seeds per hole, plant density and plants per stake in any of the seasons.

In the third season only five out of 20 farmers planted, and only three had planted climbing beans in all three seasons. About 40% did not plant in any of the seasons. From the three farmers who planted all three seasons, one farmer applied several practices (sole cropping, number of seeds per hole) consistently throughout the seasons. The other four farmers who planted in the third season switched practices (and planting of climbing beans) between seasons. The analysis over time therefore showed that the use of practices was often inconsistent and not necessarily incremental.

## 4 Discussion

### 4.1 Differences in climbing bean cultivation

Climbing bean cultivation differed between districts (Fig. 1, Table 7): 80–95% of the farmers in Kabale and Kanungu planted climbing beans in the season after the adaptation trials, but only half of the farmers in Kapchorwa. These differences point to the influence of a mixture of agro-ecological and socio-economic factors. First, farmers mentioned staking as an important constraint in Kapchorwa. The availability of trees for staking is poor in Kapchorwa district compared with Kabale and Kanungu (cf. Table 5). This is the result of a larger population pressure and more severe deforestation in Kapchorwa. Farmers in Kapchorwa were allowed regulated access to Mt Elgon forest, but at the time of study the agreement just had to be renewed and the forest was temporarily closed off which exacerbated problems of access to stakes. Especially in Kanungu, farmers often owned plantations of *Eucalyptus* or *Grevillea* where they (and their neighbours) can easily extract stakes.

Second, farmers in Kabale and Kanungu in southwestern Uganda already had a longer history of climbing bean cultivation (Table 1). This is related to the work of organisations such as the Pan-African Bean Research Alliance (PABRA) (Buruchara et al., 2011), Uganda’s National Agricultural Research Organisation (NARO), and the Association for Strengthening Agricultural Research in Eastern and Central Africa (ASARECA) (Mcharo and Katafiire, 2014), focusing on the dissemination of new varieties, seed systems and the organisation of producer groups. Southwestern Uganda has become the main production area of climbing beans in Uganda. The same organisations have worked with climbing beans in eastern Uganda, but mainly on the western instead of the northern slopes of Mt Elgon where Kapchorwa district is situated. The shorter history of climbing bean cultivation in Kapchorwa also led to a lack of seed of the distributed varieties, which made the continuation of climbing bean cultivation more difficult than in southwestern Uganda. Lack of seed is an often cited problem particularly with legume crops (David et al., 2002, Shiferaw et al., 2008).

Differences in climbing bean cultivation within districts were related to household characteristics and farmers’ previous experience with climbing bean cultivation. Both during and after the adaptation trials, household characteristics that are often associated with poorer farmers had a positive relationship with climbing bean cultivation. This is in line with earlier findings in Rwanda (Sperling and Muyaneza, 1995): although climbing beans require a considerable investment in capital and labour for staking and such investments often lead to use by wealthier farmers (Grabowski et al., 2016, Marenya and Barrett, 2007, Pircher et al., 2013), climbing beans are considered beneficial for poorer farmers because their yield potential allows intensification on small pieces of land. The more frequent planting of climbing beans by farmers who had already grown climbing beans before may indicate that farmers first need to find a specific ‘niche’ in time and space within their farm, try the beans out for a few seasons and then decide whether to continue growing them (Hockett and Richardson, 2016, Sperling and Loevinsohn, 1993).

Finally, we expected that improvements in yield in the adaptation trials resulting from the use of the improved production practices would encourage farmers to plant climbing beans in the season after. However, we observed a large variability in yield, and farmers’ own climbing bean plots yielded better than the N2Africa plots in some seasons and sites. This might lead to questions about the suitability of the technology for the area, as ‘biophysical relevance’ is the most frequently mentioned factor influencing the adoption of legumes (Farrow et al., 2016). However, variability in yields and responses to the different practices is common in on-farm trials (Franke et al., 2016, Ronner et al., 2016, Van Vugt et al., 2017). Moreover, responses to practices in the demonstrations and good yields on farmers’ own fields indicate that the technology can perform well. Late planting of the N2Africa plot is a more likely cause for the lack of response, and reflects the logistical challenges for timely supply of inputs in large-scale projects like N2Africa. Late planting probably also explained other problems referred to by farmers: pests and diseases, and destruction by stray animals that are normally tied early in the season when everybody plants. According to our analysis, trial performance did not affect farmers’ decisions to plant climbing beans in the season after the trial.

### 4.2 Differences in use of practices

The use of practices widely differed between seasons and districts. Relationships with farm size, labour, education, gender, access to credit and land tenure – common determinants of adoption (Doss, 2006, Feder and Umali, 1993) – were found. Only farm size had a consistent, positive relationship with a number of practices. Access to labour and higher education levels were expected to be positively related to the use of practices as well (Mugwe et al., 2009, Pender and Gebremedhin, 2008, Snapp et al., 2002a), but results were mixed (cf. Knowler and Bradshaw, 2007). Male farmers generally used practices more often than female farmers, which is in line with many other studies (Doss, 2001, Peterman et al., 2014, Pircher et al., 2013) and suggests that male farmers have better access to household resources. Only organic fertilizer was used more often by women farmers and female headed households, in contrast to findings of Ndiritu et al. (2014).

Relationships with household characteristics that could serve as proxies for wealth or access to credit (e.g. farm size, livestock ownership, off-farm employment and income from salary, pension or remittances) were again contrasting and inconsistent between seasons. As farmers changed practices from season to season, the latter is not surprising. Similar conclusions were drawn by Hockett and Richardson (2016), Marenya and Barrett (2007) and Misiko and Tittonell (2011): farmers experiment for a few seasons and rapidly change between practices based on performance or seasonal variations in weather, pests and diseases and access to resources. These changes may also be related to the nature of the practices that we studied: unlike investments in e.g. soil and water conservation, decisions on variety, use of fertilizer or plant density can be made on a seasonal basis. It also explains the limited relationship with land tenure, often found in studies related to longer term investments in soil improvement (Besley, 1995, Kerr et al., 2007). The lack of relationship between availability of trees for staking and stake density and length was surprising, but it may be that farmers with poor stake availability did not plant at all.

The inconsistency in use of practices over seasons contrasts with the common assumption that farmers increase the use of practices over time (Byerlee and De Polanco, 1986, Leathers and Smale, 1991) and gradually move towards adoption of the researcher best-bet. Although we found that farmers with previous experience used practices more often, this may rather be related to ‘path dependence’ – the use of practices may be dependent on earlier choices (Cowan and Gunby, 1996). Farmers who have already invested in stakes will find it easier to plant climbing beans again, or the other way around: switching to a new variety will be difficult when few farmers are growing the new variety and there is no market yet. The latter was reflected in farmers’ poorer evaluation of the marketability of the new varieties. This seemed to be a temporary problem, however, as farmers indicated in later visits that market demand for the improved varieties had increased.

Finally, similar to findings in Feder and Umali (1993), Kassie et al. (2015) and Marenya and Barrett (2007), the use of practices was often interrelated. The only practices that were complementary both during and after the adaptation trials were row planting and sole cropping, and plant density and the number of seeds per hole. Farmers who intercrop climbing beans with coffee or banana will often plant wherever there is space, so sole cropping appeared better suitable for row planting.

### 4.3 Implications for technology re-design

Farmers used different combinations of practices, and only 1% of the farmers copied the full researcher best-bet technology. In other words, 99% of the farmers adapted the technology in one way or another. This is comparable to uptake of other complex technologies like Conservation Agriculture, where farmers also adopted only components of the technology, and adaptations were not consistent among farmers (Andersson and D'Souza, 2014, Baudron et al., 2007, Pedzisa et al., 2015).

Some adaptations related to the cultivation of climbing beans by poorer farmers. For instance, farmers with smaller farms and less livestock applied the improved variety and organic fertilizer less frequently, and farmers who relied mostly on farm income and did not have income from salary, pension or remittances used fewer and shorter stakes. These adaptations hold important information that can inform the re-design of technologies (Collinson, 2000, Hockett and Richardson, 2016, Versteeg et al., 1998), and of the technology development process (Pircher et al., 2013; Tadesse et al., 2017). Composing a ‘basket of options’ suitable for farmers of different wealth and access to resources will be more useful than offering ‘fixed’ technology packages. For instance, the farmers who continued cultivating the distributed climbing bean varieties largely used them without fertilizer. This makes a comparison of local and improved varieties, both grown with and without fertilizer, a better basis for decision for farmers than a demonstration trial with improved varieties with fertilizer only (cf. Falconnier et al., 2017). The fact that many farmers grew climbing beans in intercropping instead of sole cropping may require the assessment of varieties in intercropping, which could result in breeding of varieties for intercropping conditions (Isaacs et al., 2016), tailored fertilizer recommendations for intercropping in relatively well-managed home gardens versus sole cropping on less fertile outfields (Vanlauwe et al., 2014), or specific management recommendations such as pruning of banana to enhance light availability for climbing beans (Ntamwira et al., 2013). The testing of and feedback on these options by farmers is an important part of the re-design process and helps to increase the relevance of the technology for its users (Falconnier et al., 2017, Isaacs et al., 2016, Misiko and Tittonell, 2011). Our study revealed, for instance, why some options such as the alternative staking methods were rarely used: strings were considered more expensive and labour intensive than single stakes so it turned out that strings were not ideal for poorer farmers after all.

### 4.4 Implications for recommendation domains and measurement of adoption

Understanding the diversity in climbing bean cultivation and the use of practices can be useful for the development of recommendation domains (a group of farmers with similar circumstances eligible for the same recommendation, Harrington and Tripp, 1984). These domains can be used for outscaling of technologies and the prediction of success among different groups of farmers. Based on our study and the differences between eastern and southwestern Uganda we could delineate broad domains related to tree cover, population pressure and opportunities for off-farm employment to suggest areas that are more or less likely to achieve high adoption rates of climbing beans. Within these domains, we found some significant relationships with household characteristics: poorer farmers cultivated climbing beans more often but used fewer of the best-bet practices, and male farmers generally used more practices than female farmers. Other relationships were variable or inconsistent, however, and farmers changed practices from season to season. This diversity questions the practical applicability of recommendation domains for specific farm types. Rather, it confirms the relevance of developing a ‘basket of options’ from which farmers can choose.

The diversity in use of practices also underlines the argument that adoption is not a linear, dichotomous or “once-and-for-all” process (Glover et al., 2016). For understanding the adoption process, the dynamics (i.e. through panel studies, Doss, 2006), and adaptations or different intensities of adoption (Brown et al., 2017, Glover et al., 2016, Pedzisa et al., 2015) provide more valuable information than a cross-section of farmers surveyed at one point in time. Moreover, the large variability in yields (Fig. 2, Fig. 3) illustrates that measuring impact or returns on investment is even more complicated than measuring adoption rates.

## 5 Conclusion

An average of 70% of the farmers continued the cultivation of climbing beans in the season after participation in an adaptation trial. Poor weather conditions and a lack of stakes or seed were the most frequently mentioned reasons for discontinuation of climbing bean cultivation, of which only the lack of stakes can be considered a negative attribute of the technology itself. Staking is a common constraint for climbing bean cultivation, and although alternative staking materials were demonstrated to farmers in this study, their poor uptake does not suggest that this constraint can easily be overcome. The lack of seed requires specific attention for seed systems for (improved) climbing bean varieties.

Late planting reduced the performance of the adaptation trials and reflects logistical challenges associated with large-scale dissemination projects. Trial performance did not seem to affect climbing bean cultivation or the use of practices, however. Differences between districts including tree cover, population pressure and opportunities for off-farm income played a more important role and could be used as basis for broad recommendation domains for the cultivation of climbing bean. Differences within districts and inconsistent relationships with household characteristics complicated the prediction of use of practices among farmers. This warrants the development of a basket of options from which farmers may select the practices that they consider most relevant for their particular circumstances in any given season. Our results show how adoption of technologies consisting of multiple components is a complicated process that is hard to capture through the measurement of an adoption rate at one point in time.

## Footnotes

1 Household characteristics for farmers who did not plant the adaptation trial only available in season 2014B; in 2015A and 2015B only collected for farmers who planted the trial. Results presented for adaptation trial are for season 2014B only.

2 Kapchesombe and Kaptanya sub-counties.
