# Supplementary material for: Farmers’ use and adaptation of improved climbing bean production practices in the highlands of Uganda
Source: Agric Ecosyst Environ. 2018 Jul 1;261:186–200. doi: 10.1016/j.agee.2017.09.004 (PMC5946703; doi:10.1016/j.agee.2017.09.004)
Supplement: Supplementary file 1 [file mmc1.docx]

*Supplementary material: Farmers’ use and adaptation of improved climbing bean production practices in the highlands of Uganda*

**Table S1: Varieties, inputs, staking and planting methods shown in demonstration trials in Kabale, Kanungu and Kapchorwa Districts per season**

| Variety | Cattle manure | TSP | DAP | Staking method | Planting method | Kabale and Kanungu | Kapchorwa | Source of practice |
| --- | --- | --- | --- | --- | --- | --- | --- | --- |
| NABE 12C | - | - | - | Single stakes | Row planting | 2015A, 2015B | 2014B, 2015A, 2015B | Research |
| NABE 12C | + | - | - | Single stakes | Row planting | 2015A, 2015B | 2014B | Research |
| NABE 12C | - | + | - | Single stakes | Row planting | 2015A, 2015B | 2014B, 2015A, 2015B | Research |
| NABE 12C | + | + | - | Single stakes | Row planting | 2015A, 2015B | 2014B, 2015A, 2015B | Research |
| NABE 12C | - | - | + | Single stakes | Row planting | NA | 2015A, 2015B | Co-design |
| Local Kabale | - | - | - | Single stakes | Row planting | NA | 2014B | Research |
| Local Kabale | + | - | - | Single stakes | Row planting | NA | 2014B | Research |
| Local Kabale | - | + | - | Single stakes | Row planting | NA | 2014B | Research |
| Local Kabale | + | + | - | Single stakes | Row planting | NA | 2014B, 2015A, 2015B | Research |
| NABE 10C | + | + | - | Single stakes | Row planting | NA | 2015A, 2015B | Co-design |
| Nabe 26C | - | - | - | Single stakes | Row planting | 2015A | NA | Research |
| NABE 26C | + | + | - | Single stakes | Row planting | 2015A | 2015A | Research |
| Fe-enriched | + | + | - | Single stakes | Row planting | 2015A, 2015B | 2015A, 2015B | Research |
| Fe-enriched | - | - | - | Single stakes | Row planting | 2015A, 2015B | NA | Research |
| Fe-enriched | - | + | - | Single stakes | Row planting | 2015B | NA | Research |
| Katuna | - | - | - | Single stakes | Row planting | 2015A, 2015B | NA | Co-design |
| Katuna | - | + | - | Single stakes | Row planting | 2015B | NA | Co-design |
| Katuna | + | + | - | Single stakes | Row planting | 2015A, 2015B | NA | Co-design |
| NABE 12C | + | + | - | Tripods | Row planting | NA | 2014B | Co-design |
| NABE 12C | + | + | - | Strings | Row planting | 2015A, 2015B | 2014B | Co-design |
| NABE 12C | - | + | - | Tripods | Row planting | NA | 2015A, 2015B | Co-design |
| NABE 12C | - | + | - | Strings | Row planting | NA | 2015A, 2015B | Co-design |
| Local Kabale | + | + | - | Tripods | Row planting | NA | 2014B | Co-design |
| Nabe 12C | + | + | - | Single stakes | Broadcasting | 2015A, 2015B | NA | Co-design |
